# Point-of-Care Ultrasound to Evaluate a Teenager with Presyncope

**DOI:** 10.5811/westjem.2015.12.28922

**Published:** 2016-03-02

**Authors:** Michael T. Long, Samuel Lam

**Affiliations:** Rady Children’s Hospital, Division of Emergency Medicine, San Diego, California University of California at San Diego, Department of Emergency Medicine, San Diego, California

A 16-year-old male presented with three months of palpitations at rest, fatigue, and episodic pre-syncope; his paternal grandfather died following presumed premature myocardial infarction at age 30. He was seen and discharged one week previously at an outside emergency department (ED). He followed up with his pediatrician and was promptly referred to our pediatric ED for evaluation given his risk factors. Pertinent vitals on arrival were pulse 110, blood pressure 129/66, and oxygen saturation 97% on room air. His exam was remarkable for a left upper sternal border 2/6 holosystolic murmur with radiation to apex. In addition, the patient had a chest radiograph (Figure), a nonspecific but abnormal EKG, and a point-of-care ultrasound (POCUS) of the heart performed.

POCUS (Video) suggested hypertrophic cardiomyopathy (HCM); this was confirmed by comprehensive echocardiogram that showed 4.7cm septal hypertrophy with intraventricular obstruction. Patient was admitted to the cardiac intensive care unit; during admission non-sustained runs of ventricular tachycardia were recorded. A pacemaker was placed before he was discharged on metoprolol and aspirin.

HCM has a prevalence of 1 in 500 and should be suspected with septal thickness ≥15mm or with other left ventricular or apical hypertrophy.^1^,^2^ If performed, M-mode imaging may demonstrate systolic anterior mitral valve motion, which is specific for HCM. Our patient’s diagnosis was not immediately apparent. Performance of POCUS helped confirm the diagnosis, risk-stratify the patient rapidly, and obtain timely consultation and disposition.

## Figures and Tables

**Figure:**
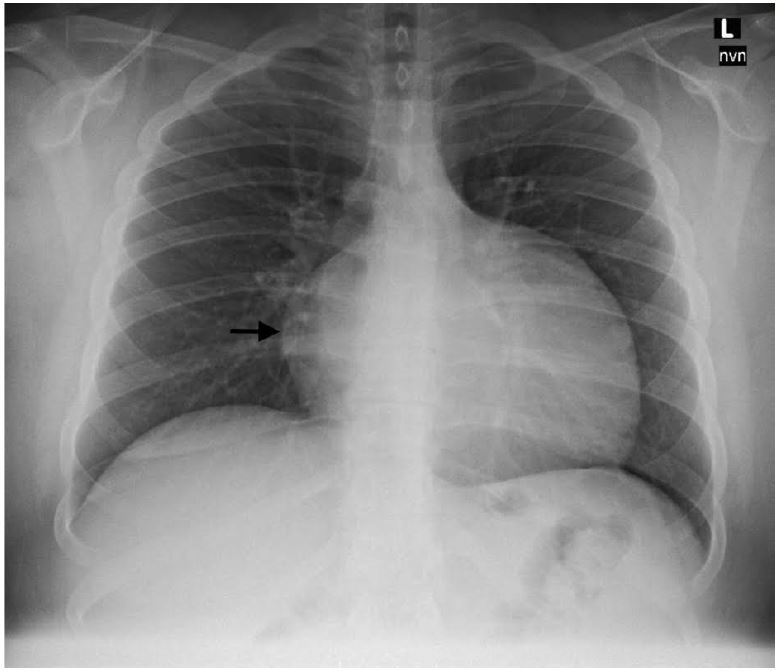
Chest radiograph showing enlarged cardiomediastinal silhouette with narrow vascular pedicle.

**Video:** Point-of-care ultrasound, cardiac views.
